# Survival benefit of local treatment for oligo-recurrence after esophageal cancer surgery

**DOI:** 10.1186/s12957-026-04212-x

**Published:** 2026-01-24

**Authors:** Katsushi Takebayashi, Sachiko Kaida, Reiko Otake, Asuka Fukuo, Toru Miyake, Masatsugu Kojima, Soichiro Tani, Hiromitsu Maehira, Haruki Mori, Nobuhito Nitta, Masaji Tani

**Affiliations:** 1 Department of Surgery, Shiga University of Medical Science, Seta Tsukinowa-cho, Otsu, Shiga 520-2192 Japan; 2 Division of Clinical Nutrition, Shiga University of Medical Science Hospital, Seta Tsukinowa-cho, Otsu, Shiga 520-2192 Japan

**Keywords:** Oligo, Local treatment, Postoperative recurrence, Esophageal cancer, Surgical resection

## Abstract

**Background:**

Postoperative recurrence of esophageal cancer is generally associated with poor survival; however, oligo-recurrence, a limited pattern of recurrence, may influence long-term survival outcomes.

**Methods:**

We retrospectively analyzed 57 patients who developed postoperative recurrence of thoracic esophageal cancer between April 2016 and March 2024. Oligo-recurrence was defined as recurrence involving three or fewer lesions confined to a single organ. Recurrence patterns, treatment strategies, and post-recurrence survival were evaluated.

**Results:**

The cohort included 48 men and 9 women, with a median age at recurrence of 72 years (range: 50–84 years). Oligo-recurrence occurred in 23 patients (40.4%); of these, 18 (78.3%) received local treatment (surgery or chemoradiotherapy), and 5 (21.7%) received chemotherapy alone. The 2-year post-recurrence survival rate was significantly higher in the oligo group than in the non-oligo group (50.8% vs. 3.7%, *p* < 0.05). Within the oligo group, 11 patients who achieved disease control with local treatment had a 2-year survival rate of 80%, compared with 13% in the remaining patients (*p* < 0.05). Univariate analysis identified early recurrence within 1 year (hazard ratio [HR]: 1.94; 95% confidence interval [CI]: 1.014–3.811; *p* < 0.05), oligo-recurrence (HR: 0.28; 95% CI: 0.143–0.555; *p* < 0.05), and local treatment (HR: 0.23; 95% CI: 0.107–0.525; *p* < 0.05) as significant factors. In multivariate analysis, only local treatment remained independently associated with improved survival (HR: 0.31; 95% CI: 0.087–0.998; *p* = 0.048).

**Conclusions:**

Local treatment for oligo-recurrence is associated with improved survival outcomes in selected patients with recurrent esophageal cancer.

**Supplementary Information:**

The online version contains supplementary material available at 10.1186/s12957-026-04212-x.

## Introduction

The recurrence rate of esophageal cancer after curative esophagectomy ranges from 28% to 47% [1]. Although immune checkpoint inhibitors have improved outcomes for recurrent disease, the median survival time after recurrence remains limited, typically ranging from 7 to 18 months [2, 3]. Most recurrences occur within 38 months post-surgery, with nearly half developing in the first year [2].

Neoadjuvant chemotherapy, aimed at controlling both local disease and micrometastases prior to surgery, has been shown to enhance curative potential and improve survival [4]. In Japan, it is considered the standard treatment strategy for patients with locally advanced esophageal cancer [5, 6]. The JCOG1109 trial showed that docetaxel, cisplatin, and 5-fluorouracil (DCF) significantly prolonged overall survival (OS) and recurrence-free survival compared with cisplatin and 5-fluorouracil alone, establishing DCF as the standard regimen in Japan [7]. In Western countries, perioperative chemotherapy with fluorouracil, leucovorin, oxaliplatin, and docetaxel has increasingly been adopted in recent years as the standard approach for resectable esophageal cancer [8].

Metastatic esophageal cancer is generally considered incurable and is typically managed with palliative intent. However, some reports have described long-term survival and even potential cures in selected patients who underwent surgical resection of recurrent lesions, particularly those limited to the lymph nodes or lungs. While the role of local treatment for recurrent esophageal cancer (REC) remains controversial, these reports suggest that aggressive interventions may be beneficial in carefully selected cases [2, 3, 9–14]. For instance, lymphadenectomy has shown potential efficacy in cases of regional lymph node recurrence, particularly in the cervical region [9–13]. Additionally, there have been reports of successful resections of recurrent lesions in organs such as the lungs, liver, brain, parotid gland, and pancreas [14–17].

Given the uncertainty surrounding the role of surgery for REC, it is therefore essential to clarify which patients may benefit from local treatment. This study aimed to investigate the association between treatment strategies and post-recurrence survival in patients with REC, with particular attention to oligo-recurrence.

## Methods

### Patients

This single-center, retrospective cohort study included 192 consecutive patients who underwent radical esophagectomy for thoracic esophageal cancer at Shiga University of Medical Science Hospital, Japan, between April 2016 and March 2024. Detailed characteristics of the 192 patients are presented in Supplementary Tables 1 and 2. Of these, 57 patients who developed postoperative recurrence were included in the analysis.

Tumor staging and pathological classification followed the Japanese Classification of Esophageal Cancer [18]. Immunonutritional status was evaluated using the Onodera Prognostic Nutritional Index (PNI) and the Controlling Nutritional Status (CONUT) score, as previously described [19, 20]. The recurrence-free interval (RFI) was defined as the time from the date of esophagectomy to the date of first confirmed recurrence.

All procedures were approved by the Ethics Committee of Shiga University of Medical Science (registration no. R2019-143) and conducted in accordance with the Helsinki Declaration of 1964 and its later amendments. Informed consent was obtained from all patients or their family members on an opt-out basis.

### Definition of oligo-recurrence

Oligo-recurrence was defined as the presence of a limited number of metastatic lesions potentially curable with local treatment. In this study, it specifically referred to recurrence involving three or fewer lesions confined to a single organ or anatomical domain. In this study, oligo-recurrence was defined as recurrence with ≤ 3 metastatic lesions confined to a single organ. This definition was based on previously proposed concepts of oligo-recurrence and oligometastatic disease, which emphasize limited tumor burden with controlled primary disease. While previous studies have used variable criteria regarding the number and distribution of metastatic lesions, we adopted a relatively stringent definition to ensure clinical homogeneity and to identify patients who were potential candidates for aggressive local treatment with curative intent [21, 22]. Oligo-recurrence was identified in 23 of the 57 patients (40.4%) in this study.

### Follow-up after esophagectomy and diagnosis of recurrence

Patients were followed up monthly during the first 3 months after surgery, and every 3 months thereafter for the next 3 years. Each follow-up visit included a physical examination and laboratory testing. Routine surveillance included measurement of serum tumor markers and computed tomography (CT) scans from the neck to the abdomen. Additional assessments, such as endoscopy, abdominal ultrasonography, brain magnetic resonance imaging, or positron emission tomography/CT, were performed when clinically indicated.

In the present study, treatment strategies for oligo-recurrent disease were determined through a multidisciplinary tumor board involving surgeons, medical oncologists, radiation oncologists, and radiologists. The selection of surgery, chemoradiotherapy (CRT), or chemotherapy alone was based on a comprehensive assessment of several objective and semiobjective factors, including Eastern Cooperative Oncology Group performance status, technical resectability of recurrent lesions, site and number of recurrences, prior treatment history, and the anticipated tolerability of aggressive local treatment.

Surgical resection was considered for patients with good performance status, technically resectable lesions, and an acceptable risk profile based on previous treatments. CRT was selected for patients with unresectable but localized disease or when surgery was deemed inappropriate due to anatomical or functional considerations. Chemotherapy alone was chosen for patients with poor performance status, disseminated disease beyond the criteria for oligo-recurrence, or when local treatment was considered unlikely to provide clinical benefit.

### Follow-up after treatment for recurrence

Post-recurrence follow-up consisted of clinical examinations, tumor marker testing, and CT scans every 3 months for the first 2 years, after which evaluations were individualized according to recurrence risk. Among patients who underwent local treatment for recurrence, response was evaluated using CT and other imaging studies according to RECIST criteria. OS was calculated from the date of initial surgery to the date of death and analyzed using the Kaplan–Meier method.

### Statistical analysis

All analyses were performed using EZR (Saitama Medical Center, Jichi Medical University, Saitama, Japan), a graphical user interface for R (version 2.13.0; R Foundation for Statistical Computing, Vienna, Austria) [23]. Survival curves were generated using the Kaplan–Meier method, and group differences were assessed using the log-rank test and Cox proportional hazards model. Univariate and multivariate analyses were conducted to identify independent prognostic factors. Confidence intervals (CIs) were calculated at the 95% level. Bonferroni correction was applied for three pairwise comparisons. All p-values were two-sided, and values < 0.05 were considered statistically significant.

Because of the retrospective nature of the study, potential confounding by indication was considered. Although propensity score–based methods, such as matching or inverse probability of treatment weighting, were explored, the limited sample size and insufficient number of events per covariate raised concerns regarding model instability and overfitting. Therefore, we performed multivariate Cox proportional hazards regression including clinically relevant variables to adjust for potential confounders.

## Results

### Patient characteristics and clinical outcomes

Baseline characteristics of the 57 patients with postoperative recurrence are summarized in Table 1. Of these, 23 patients (40.4%) met the criteria for oligo-recurrence (oligo group), and 34 (59.6%) had non-oligo recurrence (non-oligo group). The median follow-up duration after recurrence was 24.2 months (range: 1–90 months). Demographic characteristics, tumor-related factors, comorbidities, and surgical procedures were comparable between the two groups, with no statistically significant differences. However, the RFI was significantly longer in the oligo group than in the non-oligo group (median: 10.3 vs. 6.9 months; *p* < 0.05). Median survival after recurrence was also significantly longer in the oligo group (16.4 vs. 5.8 months; *p* < 0.05).

**Table 1:** Baseline characteristics of 57 patients with recurrence, stratified by metastatic pattern

	Oligo group (*n* = 23)	Non-Oligo group (*n* = 34)	*p* value
Age (yrs), median	74 (52-84)	70 (48-84)	0.92
Sex (male / female) (n, %)	17 (73.9%) / 6 (26.1%)	31 (91.2%) / 3 (8.8%)	0.1
Histological type SCC / AC (n, %)	18 (78.3%) / 5 (21.7%)	29 (85.3%) / 5 (14.7%)	0.49
Location Ce, Ut / Mt, Lt, Ae (n, %)	3 (13%) / 20 (87%)	5 (14.7%) / 29 (85.3%)	0.91
cStage (I / II / III / IVa) (n, %)	1 (4.3%)	3 (8.8%)	0.6
	5 (21.7%)	8 (23.5%)	1
	17 (74.0%)	19 (55.9%)	
	0	4 (11.8%)	
Preoperative treatment	14 (60.9%) /	22 (64.7%) /	0.1
CT / CRT / none (n, %)	9 (39.1%) / 0	2 (5.9%) / 10 (29.4%)	7
pStage (I / II / III) (n, %)	3 (13.0%)	5 (14.7%)	0.48
	5 (21.8%)	10 (29.4%)	
	15 (65.2%)	19 (55.9%)	
Body Mass Index (kg/m^2^), median	20.7 (14.4-37.5)	20.1 (12.6-31.2)	0.22
Onodera PNI, median	42.8 (34–52.2)	40.8 (33.9–58.3)	0.24
Charlson Comorbidity Index	11 (47.8%) / 12	19 (55.9%) / 15	0.55
0 / ≥1 (n, %)	(52.2%)	(44.1%)	
Esophagectomy video-assisted / open (n, %)	21 (91.3%) / 2 (8.7%)	30 (88.2%) / 4 (11.8%)	0.71
Lymph node dissection 2 fields / 3 fields (n, %)	5 (21.7%) / 18 (78.3%)	10 (29.4%) / 24 (70.6%)	0.79
Reconstruction gastric tube / ileocolon (n, %)	18 (78.3%) / 5 (21.7%)	24 (70.6%) / 10 (29.4%)	0.79
RFI, months median (range)	10.3 (1-42)	6.9 (1-27)	< 0.05
Recurrence sites (n, %)	4 (17.4%)	2 (5.9%)	0.23
Local	10 (43.5%)	18 (52.9%)	
Lymph node	2 (8.7%)	7 (20.6%)	
Lung	1 (4.3%)	9 (26.4%)	
Liver	2 (8.7%)	4 (11.8%)	
Bone	0	1 (2.9%)	
Adrenal	3 (13.1%)	2 (5.9%)	
Pleura	1 (4.3%)	1 (2.9%)	
Brain			
Locoregional / distant / mixed	14 (60.9%) / 9 (39.1%) / 0	10 (29.4%) / 16 (47.1%) / 8 (23.5%)	
No. of recurrent tumors			< 0.05
1	6	0	
2	17	3	
≥3	0	31	
Treatment for the recurrence			
Surgery	7 (30.4%)	0	< 0.05
CRT	11 (47.9%)	5 (14.7%)	
CT	5 (21.7%)	29 (85.3%)	
Survival time after recurrence, months, median (range)	16.4 (3-82)	5.8 (1-37)	<0.05

*Oligo* Oligo-metastasis, *SCC* Squamous cell carcinoma, *AC* Adenocarcinoma, *cStage* Clinical stage, *CT* Chemotherapy, *CRT* Chemoradiotherapy, *pStage* Pathological stage, *PNI* Prognostic nutritional index, *RFI* Recurrence-free interval

### Treatment strategies and outcomes by recurrence site

Treatment outcomes are shown in Table 2. In the oligo group, 18 patients (78.3%) received local treatment (surgery or CRT), and 5 (21.7%) received systemic chemotherapy alone. Surgical resection was performed in 7 patients, with R0 resection achieved in 6 (85.7%). Pleural dissemination was resected locally, and no adjuvant whole-brain irradiation was administered for brain metastases. No patients in the non-oligo group underwent surgery.

**Table 2:** Treatment strategies and outcomes of recurrences, stratified by metastatic site

	Oligo group (n = 23)	Non-Oligo group (n = 34)	*p* value
Surgery (n = 7)			< 0.05
Local	n = 7	n = 0	
Lymph node	1	0	
Lung	2	0	
Liver	1	0	
Bone	0	0	
Adrenal	0	0	
Pleura	0	0	
Brain	2	0	
	1	0	
Radicality			
R0 / R1 (n, %)			
Postoperative	6 (85.7%) / 1 (14.3%)	-	
Complications	1 (wound infection)	-	
CRT (n = 17)	n = 11	n =5	
Local (CR)	3 (0)	2 (0)	
Lymph node (CR)	6 (3)	2 (0)	
Lung (CR)	1 (1)	1 (0)	
Liver (CR)	0	0	
Bone (CR)	0	0	
Adrenal (CR)	0	0	
Pleura (CR)	1 (1)	0	< 0.05
Brain (CR)	0	0	
Response rate (%)	90.9%	80%	0.54
CR / PR / SD / PD	5 / 5 / 0 / 1	2 / 2 / 0 / 1	
Adverse event			
Radiodermatitis	5	2	0.83
Neutoropenia	5	2	0.83
Radiation pneumonitis	1	0	0.44
CT (n = 34)	n = 5	n = 29	
Local (CR)	1 (0)	2	
Lymph node (CR)	2 (0)	15 (0)	
Lung (CR)	0	4 (0)	
Liver (CR)	0	10 (0)	
Bone (CR)	1 (0)	2 (0)	
Adrenal (CR)	1 (0)	1 (0)	
Pleura (CR)	0	3 (0)	
Brain (CR)	0	1 (0)	< 0.05
Response rate (%)	40%	34.5%	
CR / PR / SD / PD	0 / 2 / 2 / 1	0 / 10 / 15 / 4	0.81
Adverse event			
Neutropenia	2	6	0.34
Anemia	1	3	0.53
Thrombocytopenia	1	3	0.53
Nausea and vomiting	1	5	0.88
Loss of appetite	3	12	0.43
Mucositis	1	5	0.88
Diarrhea	1	4	0.71
Peripheral neuropathy	1	3	0.53

*CRT* Chemoradiotherapy, *CR* Complete response, *PR* Partial response, *SD* Stable disease, *PD* Progressive disease, *CT* Chemotherapy

The response rate to CRT was higher in the oligo group than in the non-oligo group (90.9% vs. 80.0%), although the difference was not statistically significant (*p* = 0.54). The response rate to chemotherapy alone was low in both groups (40.0% vs. 34.5%; *p* = 0.81). In addition, we found that postoperative complications and chemotherapy and CRT-related adverse events did not have a significant impact on overall survival in our cohort. Of the 7 patients who underwent surgical resection, 3 subsequently received systemic chemotherapy, and of the 16 patients who received CRT, 5 were later treated with systemic chemotherapy.

### Survival outcomes according to recurrence pattern and treatment

**Fig. 1:**
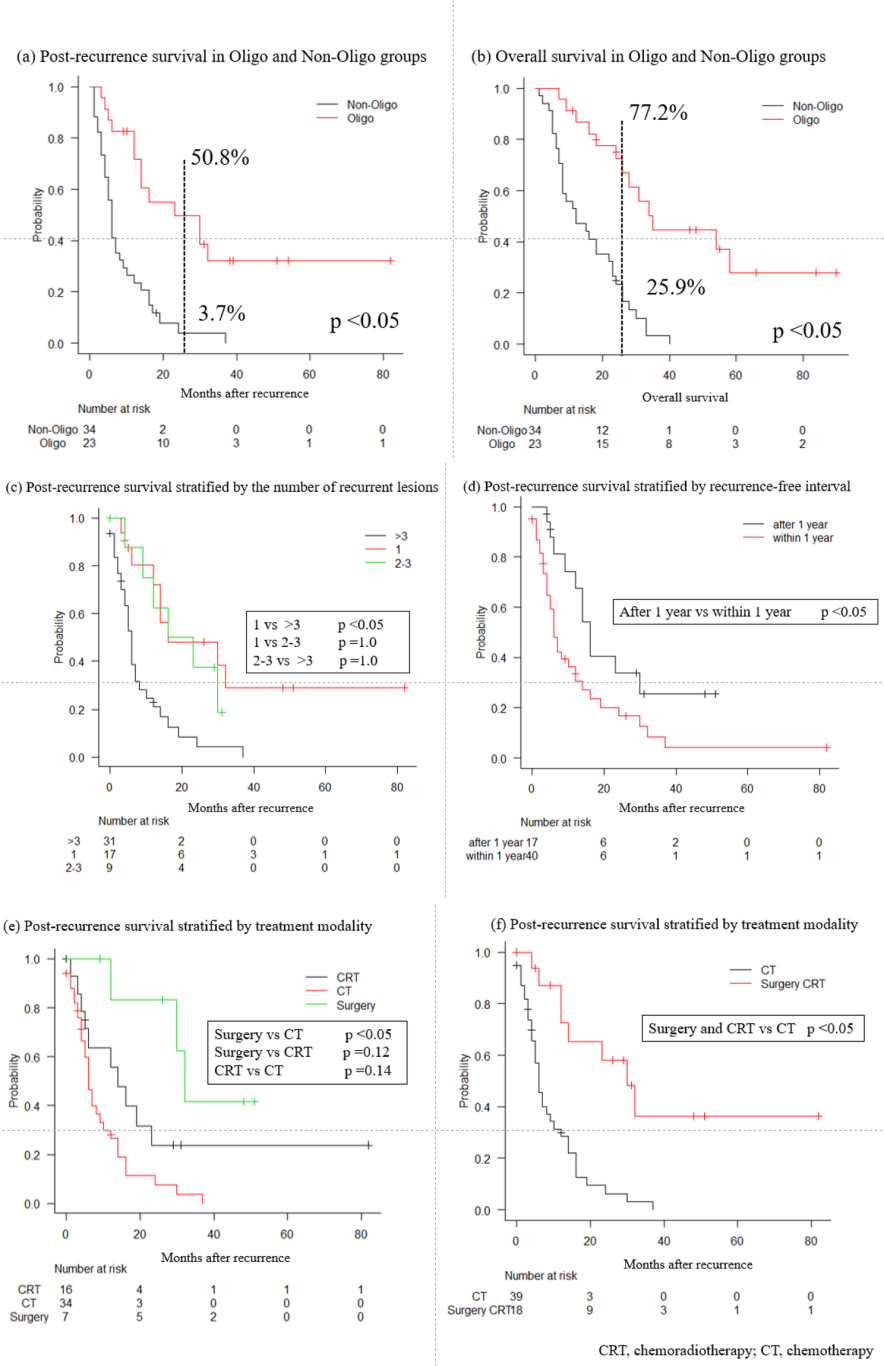
Post-recurrence survival by recurrence pattern, lesion number, recurrence-free interval, and treatment modality. *p*-values were calculated using the log-rank test, with Bonferroni correction for three pairwise comparisons. **a** Kaplan–Meier curves comparing post-recurrence survival between the oligo and non-oligo groups. **b** Overall survival from initial surgery in the oligo and non-oligo groups. **c** Post-recurrence survival stratified by the number of recurrent lesions. **d** Post-recurrence survival by recurrence-free interval. **e**, **f** Post-recurrence survival by treatment modality

Figure 1 illustrates survival outcomes after recurrence, stratified by recurrence pattern, number of recurrent lesions, RFI, and treatment strategies. As shown in Fig. 1a, the 2-year post-recurrence survival rate was significantly higher in the oligo group than in the non-oligo group (50.8% vs. 3.7%; *p* < 0.05). Similarly, OS from the initial surgery was significantly longer in the oligo group (77.2% vs. 25.9%; *p* < 0.05) (Fig. 1b).

Survival outcomes based on the number of recurrent lesions are shown in Fig. 1c. Patients with a single lesion had significantly better post-recurrence survival than those with more than three lesions (*p* < 0.05). Fig. 1d shows that patients who experienced recurrence after 1 year had significantly better survival than those who recurred within 1 year (*p* < 0.05).

Survival according to treatment modality is presented in Fig. 1e. Patients who underwent surgery had significantly better post-recurrence survival than those who received chemotherapy alone (*p* < 0.05). When surgery and CRT were combined as local treatments, they were associated with significantly better post-recurrence survival compared with chemotherapy alone (*p* < 0.05; Fig. 1f). It should be noted that surgery was performed only in the Oligo group. Accordingly, these survival comparisons are descriptive in nature and should be interpreted with caution.

### Prognostic factors for post-recurrence survival

Univariate and multivariate analyses of potential prognostic factors are shown in Table 3. In univariate analysis, early recurrence within 1 year (HR: 0.94; 95% CI: 1.014–3.811; *p* = 0.046), oligo-recurrence (HR: 0.28; 95% CI: 0.1427–0.555; *p* < 0.01), and local treatment (surgery or CRT vs. chemotherapy alone; HR: 0.24; 95% CI: 0.107–0.525; *p* < 0.01) were significantly associated with improved post-recurrence survival. In multivariate analysis, only local treatment remained an independent prognostic factor for OS after recurrence (HR: 0.31; 95% CI: 0.087–0.998; *p* = 0.048).

**Table 3:** Multivariate analysis of prognostic factors for overall survival after recurrence

Factors	HR	95% CI	Univariate analysis	HR	95% CI	Multivariate analysis
Age (> 75)	0.6022	0.3139–1.155	0.1271			
Sex (female)	0.9497	0.3964–2.276	0.9079			
cStage (I-II)	0.998	0.9744–1.021	0.844			
Histological type (SCC)	0.9923	0.992–1.015	0.503			
Body mass index (< 20)	0.991	0.9686–1.015	0.458			
Onodera PNI (< 40)	0.999	0.9898–1.054	0.745			
Charlson Comorbidity Index (≥ 1)	0.944	0.951–1.167	0.893			
RFI < 1 year	0.94	1.014–3.811	0.046	0.9825	0.9355–1.032	0.479
Oligo (vs. Non-oligo)	0.2816	0.1427–0.555	< 0.01	0.7113	0.2228–2.271	0.758
Recurrence site (locoregional)	0.987	0.9642-1.01	0.274			
Surgery and CRT (vs. CT)	0.2371	0.107–0.5253	< 0.01	0.3087	0.087–0.998	0.048

*HR* Hazard ratio, *CI* Confidence interval, *SCC* Squamous cell carcinoma, *PNI* Prognostic nutritional index, *RFI* Recurrence-free interval, *Oligo* Oligometastasis, *CRT* Chemoradiotherapy, *CT* Chemotherapy

## Discussion

In this study, we demonstrated that local treatment for oligo-recurrence after esophagectomy is associated with improved survival outcomes. Surgical resection is considered the most effective form of local control; however, CRT may offer similar curative potential when CR is achieved.

Although reports on oligo-recurrence have increased in recent years, definitions vary across studies [21, 22, 24, 25]. Kang et al. examined the relationship between the number of recurrent lesions after esophagectomy and post-recurrence survival. Solitary recurrence was the most common (38.3%) and was associated with the best survival (median: 17.1 months; *p* < 0.001) [22]. Patients with two or three recurrent lesions had similar survival, both significantly better than those with four or more lesions (median: 9.4 months; *p* < 0.001) [22]. Multivariate analysis also identified the number of recurrent lesions as a significant prognostic factor [22]. Even when metastases are confined to a single organ, resection of five lesions requires careful consideration of both oncological benefit and surgical tolerance. Based on these findings and previous conceptual frameworks emphasizing limited metastatic burden and organ-confined disease [21, 22], we defined oligo-recurrence in the present study as recurrence involving three or fewer lesions confined to a single organ. However, we acknowledge that no universally accepted definition of oligo-recurrence exists, and this criterion may influence patient selection and treatment outcomes.

Surgical resection and CRT remain the main options for local control. Aggressive resection has been shown to improve survival [24, 25]. Ohkura et al. reported that, among patients with oligorecurrence, those who underwent surgery had significantly better outcomes than those who did not [25]. Moreover, patients eligible for surgery but not treated surgically had survival comparable to those with multiple metastases [25].

The efficacy of CRT in oligometastases has also been documented [26, 27]. Yamamoto et al. reported favorable outcomes in 132 patients with pulmonary oligometastases treated with CRT, achieving a 3-year local control rate of 70.2% and an OS rate of 37.5% [21]. Chen et al. showed that, even in patients with recurrent esophageal squamous cell carcinoma previously treated with definitive radiotherapy, radical reirradiation for oligo-recurrence could achieve long-term survival in approximately 20% of patients [26].

In the non-oligo group, the response to local treatment was evaluated using imaging studies, including CT scans. Local control was incomplete in some patients, which may have contributed to the lack of significant survival differences observed across treatment modalities. Radical surgery was generally not performed in this group due to extensive disease or patient condition.

Treatment selection depends on surgical resectability, patient tolerance, institutional policy, and patient preference. At our institution, we also consider additional factors such as disease-free interval, tumor biology (histologic subtype and aggressiveness), and patient functional status during multidisciplinary committee discussions to guide the choice of the most appropriate therapy for each patient. Kudou et al. found surgical resection particularly valuable in cases of limited lymph node involvement (low pathological nodal status), pulmonary metastasis, long disease-free interval, and technically resectable lesions [24]. In our study, patients achieving local control with CRT had survival outcomes comparable to those undergoing surgery, suggesting CRT may be equally effective in selected cases, though surgery may still offer superior local control.

Multivariate analysis identified the absence of severe postoperative complications (Clavien–Dindo grade < IIIa) as an independent predictor of better prognosis [24, 27, 28]. Therefore, surgical risks should be minimized. Even when technically resectable and potentially curable, surgical indications must be determined cautiously.

Sato et al. distinguished between oligo-recurrence (controlled primary tumor with limited metastases) and sync-oligometastases (active primary tumor with limited metastases) [29]. In selected patients with limited metastases, local treatments such as surgery or definitive CRT may provide a potential cure [29, 30]. These findings may guide the development of new treatment strategies for oligometastatic esophageal cancer.

During the study period, immune checkpoint inhibitors were used in a subset of patients with recurrent esophageal cancer. Importantly, the proportion of patients with a history of immune checkpoint inhibitor therapy was comparable across the treatment groups, including surgery, chemoradiotherapy, and chemotherapy alone. Therefore, differences in survival outcomes among the groups are unlikely to be attributable to imbalances in immunotherapy exposure. With the increasing use of immune checkpoint inhibitors in routine clinical practice, systemic disease control is expected to further improve. In this context, aggressive local treatment for oligo-recurrence may play a complementary role by achieving durable local control, potentially enhancing long-term survival when combined with effective systemic therapies. Future studies are needed to determine the optimal integration and sequencing of immune checkpoint inhibitors and local treatment modalities in patients with oligometastatic or oligo-recurrent esophageal cancer.

This study has several important limitations. First, its retrospective, single-center design with a limited sample size and a relatively small number of outcome events inherently increases the risk of selection bias and confounding by indication. Treatment strategies were determined based on the discretion of the attending physicians and patient preferences, which may have influenced treatment allocation. Although propensity score–based methods or more robust multivariate modeling could theoretically mitigate this issue, the limited number of events per covariate in the present cohort precluded their reliable application, as such approaches could have resulted in model instability or overfitting.

In addition, surgery was performed exclusively in the oligo-recurrence group, further introducing potential selection bias. Therefore, the results of multivariate analyses and survival comparisons should be interpreted as exploratory and hypothesis-generating, rather than as evidence of causal relationships. Residual confounding cannot be completely excluded, and the findings should be interpreted with caution.

Nevertheless, the data were derived from a prospectively maintained database of consecutive patients and collected over a relatively short study period, which may have helped minimize information bias and ensure data consistency.

## Conclusions

Our findings suggest that local treatment for oligo-recurrence after esophagectomy is associated with improved survival outcomes.

## Supplementary Information

Supplementary Material 1.

Supplementary Material 2.

## Data Availability

The datasets used and/or analyzed during the current study are available from the corresponding author on reasonable request.
