# Supplementary material for: Survival benefit of local treatment for oligo-recurrence after esophageal cancer surgery
Source: World J Surg Oncol. 2026 Jan 24;24:93. doi: 10.1186/s12957-026-04212-x (PMC12924590; doi:10.1186/s12957-026-04212-x)
Supplement: Supplementary file 1 — Supplementary Material 1. [file 12957_2026_4212_MOESM1_ESM.docx]

Supplementary Table 1. Patient characteristics and postoperative recurrence among 192 consecutive esophagectomy cases

|  | (n =192) |
| --- | --- |
| Age (yrs), median | 70 (38-85) |
| Sex (male / female) (n, %) | 162 (84.4%) / 30 (15.6%) |
| Histological type SCC / AC  (n, %) | 170 (88.5%) / 22 (11.5%) |
| Location Ce, Ut / Mt, Lt, Ae  (n, %) | 25 (13%) / 167 (87%) |
| cStage (I / II / III / IVa) (n, %) | 48 (25%)  50 (26%)  86 (44.8%)  8 (4.2%) |
| Preoperative treatment  CT / CRT / none (n, %) | 90 (46.9%) / 10 (5.2%) / 92 (47.9%) |
| pStage (I / II / III) (n, %) | 88 (45.8%)  44 (22.9%)  60 (31.3%) |
| Body Mass Index (kg/m^2^), median | 20.3 (12.4-37.5) |
| Onodera PNI, median | 45.8 (31–53.2) |
| Charlson Comorbidity Index  0 / ≥1 (n, %) | 91 (47.4%) / 101 (52.6%) |
|  |  |
| Esophagectomy  video-assisted / open (n, %) | 162 (84.4%) / 30 (15.6%) |
| Lymph node dissection  2 fields / 3 fields (n, %) | 63 (32.8%) / 129 (67.2%) |
| Reconstruction  gastric tube / ileocolon (n, %) | 167 (87%) / 25 (13%) |
|  |  |

Oligo, oligo-metastasis; SCC, squamous cell carcinoma; AC, adenocarcinoma; cStage, clinical stage; CT, chemotherapy; CRT, chemoradiotherapy; pStage, pathological stage; PNI, prognostic nutritional index; RFI, recurrence-free interval.
