# Supplementary material for: Survival benefit of local treatment for oligo-recurrence after esophageal cancer surgery
Source: World J Surg Oncol. 2026 Jan 24;24:93. doi: 10.1186/s12957-026-04212-x (PMC12924590; doi:10.1186/s12957-026-04212-x)
Supplement: Supplementary file 2 — Supplementary Material 2. [file 12957_2026_4212_MOESM2_ESM.docx]

Supplementary Table 2. Pathological stage distribution and recurrence rates among 192 consecutive patients undergoing radical esophagectomy

| Pathological stage | Total (n=192) | Recurrence (n=57) | Recurrence rate (%) |
| --- | --- | --- | --- |
| pStage I | 88 | 8 | 9.1 |
| pStage II | 44 | 15 | 34.1 |
| pStage III | 60 | 34 | 56.7 |
|  |  |  |  |

pStage, pathological stage.
